# Patterns and Trends in Pharmacological Treatment for Outpatients with Postherpetic Neuralgia in Six Major Areas of China, 2015–2019

**DOI:** 10.3390/healthcare11050764

**Published:** 2023-03-06

**Authors:** Gang Han, Yun Han, Lingyan Yu, Yuhua Zhao, Zhenwei Yu

**Affiliations:** 1 Sir Run Run Shaw Hospital, College of Medicine, Zhejiang University, Hangzhou 310016, China; 2 Research Center for Clinical Pharmacy, Zhejiang University, Hangzhou 310058, China; 3 College of Pharmaceutical Science, Zhejiang University, Hangzhou 310058, China; 4 Second Affiliated Hospital, College of Medicine, Zhejiang University, Hangzhou 310009, China; 5 Affiliated Xiaoshan Hospital, Hangzhou Normal University, Hangzhou 311202, China

**Keywords:** gabapentin, pregabalin, oxycodone, prescription, cost

## Abstract

The aim of this study was to assess the patterns and trends of pharmacological treatment for outpatients with postherpetic neuralgia (PHN) in China in the period 2015–2019. Prescription data for outpatients with PHN were extracted from the database of the Hospital Prescription Analysis Program of China according to the inclusion criteria. The trends in yearly prescriptions and corresponding costs were analyzed and stratified by drug class and specific drugs. A total of 19,196 prescriptions from 49 hospitals in 6 major regions of China were included for analysis. The yearly prescriptions increased from 2534 in 2015 to 5676 in 2019 (*p* = 0.027), and the corresponding expenditures increased from CNY 898,618 in 2015 to CNY 2,466,238 in 2019 (*p* = 0.027). Gabapentin and pregabalin are the most commonly used drugs for PHN, and more than 30% of these two drugs were combined with mecobalamin. Opioids were the second most frequently prescribed drug class, and oxycodone accounted for the largest share of the cost. Topical drugs and TCAs are rarely used. The frequent use of pregabalin and gabapentin was in accordance with current guidelines; however, the use of oxycodone raised concerns about rationality and economic burden. The results of this study may benefit the allocation of medical resources and management for PHN in China and other countries.

## 1. Introduction

Postherpetic neuralgia (PHN) is a chronic complication of herpes zoster (HZ) caused by damage to peripheral nerve tissue during the onset of herpes zoster, which is defined as obvious pain 3 months after herpes zoster [1]. PHN is often described as burning pain, tingling or itching, and its pain score is always ≥4 on a 10-point visual analog scale [2,3]. The burning and allodynia pain of PHN in the thoracolumbar region are more intensive, while the tingling and numbness of PHN in the face are more intense [4]. Approximately 20% of herpes zoster patients develop PHN [5]. In the United States, the total incidence rate of PHN is 57.5 cases per 100,000 person-years [6]. A study on herpes zoster and its complications in China reported that the incidence rate of PHN was 0.48 per 1000 person-years [7]. The pain caused by PHN is often unbearable, which impairs the work of many employees. This is equivalent to an annual indirect loss of CNY 28,025 (USD 4221), which not only leads to the loss of personal wages but also has a wider economic impact on society as a whole through productivity loss [8,9].

There are many treatment options for PHN, mainly including the most widely used pharmacological therapy and nonpharmacological methods such as nerve blocks, neuromodulation and nerve stimulation [10,11]. In the field of pharmacological treatment, some anticonvulsants, antidepressants, opioids and local therapeutic drugs have been proven to be able to relieve pain. Among the drug recommendations in many countries, gabapentin, pregabalin and TCAs are often used as first-line drugs, while topical drugs and opioids are also often suggested in treatment [1,10,12]. At present, we have known that many drugs, such as gabapentin and pregabalin, are widely used in clinical treatment [13,14,15]. However, side effects of PHN drugs are common, and some drugs, especially opioids, have a potential risk of addiction [16]. Currently, little is known about the status of PHN drug application in China. Considering the increasing prevalence of herps zoster in China [17], we designed this cross-sectional study to analyze the patterns and trends of PHN drug use, as well as its costs.

## 2. Materials and Methods

### 2.1. Study Design and Ethics

This study was a retrospective prescription-based cross-sectional study, and informed consent was waived as part of the approval. Ethical approval was obtained from the Ethics Committee of Run Run Shaw Hospital, College of Medicine, Zhejiang University (Reference Number KEYAN20210924-33).

### 2.2. Data Source and Prescription Inclusion

Prescription data were derived from the widely used database of the Hospital Prescription Analysis Cooperative Project of China for pharmaco-epidemic studies [17,18,19,20,21]. The database was initiated in 2003, and the following items of prescription were included in the database: prescription code, date of prescription issued, sex and age of patient, department of physician, hospital code, drug generic name, strength, price and cost of drug, diagnosis. Prescriptions for patients with a diagnosis of PHN were extracted, and those meeting the following criteria were included for analysis: (1) prescriptions written from 2015 to 2019; (2) prescriptions from hospitals situated in 6 major regions of China (Beijing, Shanghai, Hangzhou, Guangzhou, Chengdu and Tianjin) and participated in the program continuously; (3) prescriptions for adult outpatients (age > 18 years) diagnosed with PHN. Prescriptions with missing values were excluded from the analysis.

### 2.3. Analysis

The prescriptions for patients with PHN were represented by the number of corresponding prescriptions per year. The annual cost was the sum of the total cost of PHN patients’ prescriptions. It should be noted that inflation factor or discount rate was not considered either. The trends in annual prescriptions and expenditures were analyzed, and further stratified and illustrated by drug class and specific drugs.

The PHN-treated drugs were classified to analyzed as follows: (1) anticonvulsants, including gabapentin, pregabalin, carbamazepine, oxcarbazepine, lamotrigine, valproic acid, topiramate and other analogs; (2) antidepressants, including tricyclic antidepressants (TCAs) and other serotonin (5-HT) and norepinephrine (NE) reuptake inhibitors; (3) opioids, compound preparations containing opioids that are also classified as opioids; and (4) topical drugs, including capsaicin, lidocaine, flurbiprofen and diclofenac [11,13,14,22,23,24].

The trends in prescription numbers and costs for overall and individual drugs were assessed using the Mann–Kendall test. The trends in percentages were assessed using the log-linear test. The Wilcoxon signed rank test was used for the difference between the male and female prescription percentages. The average proportion and standard deviation of the combined use of gabapentin and pregabalin in five years were calculated. The trend package in R (version 4.2.1) software was used for statistical analysis. The statistical significance was set as *p* < 0.05.

## 3. Results

### 3.1. Demographic Characteristics of Patients and Overall Trends

**Figure 1:**
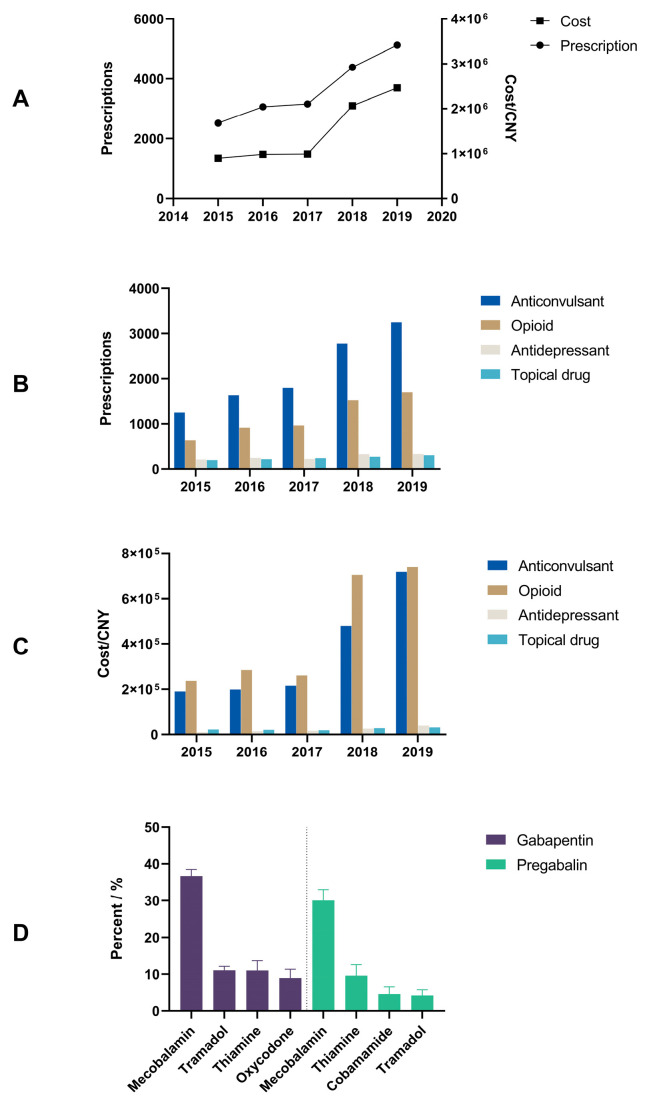
Prescription trends of PHN in six regions of China. (**A**) Annual number of patients and costs; (**B**) total prescriptions of the four classes of drug trends; (**C**) total costs of the four classes of drug trends; (**D**) average ratios of five years in the combination of gabapentin (left) and pregabalin (right). The data are expressed as the mean ± SD of 5 years.

A total of 19,196 prescriptions were included in this study. Detailed demographic characterizations of patients with PHN prescriptions are shown in Table 1. The percentages of prescriptions for females were slightly higher (*p* = 0.043), and the proportion did not significantly change during the study period (*p* = 0.198). The yearly prescriptions and expenditures are shown in Figure 1A. The yearly prescriptions increased from 2534 in 2015 to 5676 in 2019 (*p* = 0.027), and the corresponding expenditures increased from CNY 898,618 in 2015 to CNY 2,466,238 in 2019 (*p* = 0.027).

### 3.2. Trends in Prescriptions and Cost of Drug Class and Specific Drug

The yearly total prescriptions for four major classes of PHN drugs—anticonvulsants, antidepressants, opioids and topical drugs—increased during the study period (Figure 1B), and detailed prescription numbers are listed in Table 2. Anticonvulsants were the most frequently prescribed drug class, followed by opioids. Antidepressants and topical drugs were rarely used.

Table 3 shows the costs and percentage of specific drugs. There was a certain difference between the trend in expenditure and the trend in prescriptions. The total costs of opioids were always higher than those of anticonvulsants, which had most prescriptions (Figure 1C).

Gabapentin and pregabalin were the most frequently used drugs. Prescriptions of pregabalin increased rapidly (*p* = 0.002), with the largest increase of 379% in 2018. Regarding second-line opioid drugs, the proportion of oxycodone prescriptions was large and continuously increasing (*p* = 0.031). For antidepressants, the number of prescriptions of traditional TCA amitriptyline was greater than the others. The topical drugs were mainly lidocaine and capsaicin.

In anticonvulsants, the costs of pregabalin also increased. The average proportion of oxycodone costs per year was approximately 21.3% of the total costs. This was the drug with the largest proportion of the annual amount, and the proportion was stable (*p* = 0.220). Among antidepressants, the total costs of duloxetine and venlafaxine were higher, while the total costs of amitriptyline were lower.

### 3.3. Trends in Combination of Drugs

Gabapentin and pregabalin, as the first-line choice drugs, were combined with other drugs (Figure 1D). Mecobalamin was the drug most commonly used in combinations. On average, 36.7% of gabapentin prescriptions jointly used mecobalamin, as well as 30.0% of pregabalin prescriptions.

## 4. Discussion

This is the first study to analyze the patterns and trends in pharmacological treatment for outpatients with PHN in China. The yearly prescriptions and costs of PHN drugs have been increasing. Two anticonvulsant drugs—gabapentin and pregabalin—were the most commonly used drugs, which was in line with current practice guidelines. At the same time, we also found that oxycodone in opioids was used in large quantities and costs in a large proportion, which might be an unreasonable use in the treatment of PHN. The percentages of antidepressants and topical drugs were relatively low, in both prescriptions and corresponding costs. Regarding the combination of gabapentin and pregabalin, we unexpectedly found that mecobalamin was used more frequently.

The prescriptions for patients with PHN increased during the study period. In China, 7.26% of herpes zoster patients have PHN, and the incidence rate in women is slightly higher than that of men (7.45% vs. 7.03%) [7], which is consistent with the results of our study. In addition, the number of people diagnosed with herpes zoster continued to increase from 2015 to 2019 [17]. Therefore, the increase in the number of people diagnosed with PHN might be related to the progressive rise of prescriptions for PHN.

At present, the treatment of PHN is based on symptom control, and many studies have proven that antiviral drugs for herpes zoster have no significant effect on PHN or its prevention. Therefore, the treatment of PHN usually follows the principle of neuralgia treatment [25,26]. In the Chinese guidelines, the first-line drugs include pregabalin and gabapentin, TCAs (such as amitriptyline, etc.) and 5% lidocaine patches, and the second-line drugs include opioids [27]. The first-line treatment of PHN in the United States includes TCAs, gabapentin and pregabalin, and a topical lidocaine 5% patch. Opioids and capsaicin patches are recommended as second-line or third-line therapeutic drugs [14]. The French guidelines for neuralgia regard TCAs and other serotonin-norepinephrine reuptake inhibitors (duloxetine and venlafaxine, etc.), and gabapentin as first-line drugs for the treatment of neuralgia, with pregabalin, weak opioid tramadol and capsaicin patches recommended as second-line drugs, and other powerful opioids as third-line drugs [13]. According to the Canadian Pain Society consent statement, gabapentin, TCAs and serotonin-norepinephrine reuptake inhibitors are first-line drugs for the treatment of neuropathic pain. Opioids are recommended as second-line drugs, while cannabinoids are newly recommended as second-line drugs [15]. In general, gabapentin, pregabalin and TCAs are often used as first-line drugs, while opioids are not the first choice for PHN. Thus, the use of most frequently prescribed anticonvulsant, mainly gabapentin and pregabalin, was in accordance with current guidelines and evidences. Regional differences in drug use were not significant in this study.

Gabapentin and pregabalin, which are used most, are voltage-gated cation channel regulators [28,29]. Daily doses of 1800 mg to 3600 mg of gabapentin can provide patients with effective pain relief levels [30]. The general dosage of pregabalin for the treatment of PHN is between 75 mg and 600 mg per day, which is taken two to three times per day [31]. In our study, the treatment time of gabapentin single prescription is generally about 16 days, and the single oral dose is between 100 mg and 1500 mg, two to four times a day. The treatment time of pregabalin in a single prescription varies greatly, and the single oral dose is between 75 mg and 300 mg, one to three times a day. It is similar to the recommended dosage. The use of pregabalin had a significant increase during the study period, and its prescriptions exceeded gabapentin in 2018. Gabapentin and pregabalin are recognized as drugs with good relief effects on PHN [32]. The increase in the use of pregabalin may be due to the following reasons. The first is the difference in the results of drug action. Omar et al.’s study on the difference between pregabalin and gabapentin initially showed that pregabalin was better at alleviating pain, while gabapentin had better effects on anxiety, insomnia and fatigue symptoms [33]. Previous studies have confirmed that pregabalin is highly effective and safe for patients with PHN in China [34]. Additionally, the widespread use of gabapentin and pregabalin calls special focus to the effective management of its use, as these drugs also have side effects. An overdose of gabapentin and pregabalin will produce euphoric effects and can lead to delirium [35]. Compared with pregabalin, the abuse of gabapentin is a growing trend. A British survey found that the proportion of lifetime gabapentin abuse was 1.1%, compared with 0.5% in pregabalin [36,37]. Another reason for this may be related to the expiration of the patent. According to the database of the China Pharmaceutical Industry Information Center, the patent protection date of pregabalin expired in 2018. Although the brand of pregabalin used by patients has not changed in the past five years, the expiration of the patent has increased the attention it has received in wider society, especially for PHN patients, medical institutions and related pharmaceutical companies. More prescribers realize that the role of pregabalin in PHN may be better than that of gabapentin, so they are more willing to prescribe pregabalin. Other anticonvulsant drugs, such as oxcarbazepine, have proven to have no better therapeutic effect than both gabapentin and pregabalin on neuralgia, and their use is rare [38].

Opioids are widely used in pain control, and oxycodone is the drug with the largest number of prescriptions in the current study. Oxycodone is a semisynthetic μ- and κ-opioid receptor agonist with a wide range of applications [39]. Some studies have also shown that the use of oxycodone is not completely beneficial to the treatment of PHN [29,40]. A study by Gaskell et al. on oxycodone in the treatment of neuralgia suggested that there was no reported result within the scope of their study that can prove that oxycodone has substantial benefit results, such as the overall impression of clinical changes in the treatment of neuralgia [41]. Thus, although opioids were recommended as second-line treatment for PHN, oxycodone was not recommended, or only a very weak recommendation. However, oxycodone has the advantages of long duration of action and no histamine release or ceiling effect compared with other opioids, so it is still used frequently [39]. Compared with the status of other countries, the study by Gudin et al. found that 21.6% of PHN patients received opioids as initial treatment for PHN in the United States, while among the other first-line treatment methods of PHN, gabapentin was 15.1%, pregabalin was 3.3% and TCAs were 2.5%, which proved that excessive use of opioids was common [42]. Opioids are prone to cause peripheral nerve injury, which leads to increased noxious hypersensitivity, various adverse reactions and drug interactions [43,44,45]. It can also be seen from the conclusion that the cost of oxycodone accounts for a large proportion of overall expenditure and its spending has been sustained at a high level over the five years period of the study. Therefore, the widespread use of oxycodone has raised concerns about rationality and the economic burden on patients. For this phenomenon, the relevant departments should maintain a high degree of vigilance and remind prescribers to reduce or limit the use of related addictive drugs if necessary. Prescribers should evaluate the pain degree of patients before using drugs, and relevant departments can set different indicators of analgesic use for different pain levels, so as to re-evaluate whether opioid analgesics should be used to manage the PHN.

The use of antidepressants is far lower than that of anticonvulsants and opioids, which reflects physician behavior and patient preference. Antidepressants have a certain relieving effect on PHN [46]. However, TCAs such as amitriptyline cannot achieve satisfactory effects for all people in the treatment of PHN pain [47,48]. Another reason for the infrequent use of TCAs is the adverse effects of TCAs. They may cause nausea, headache, constipation and other negative effects that patients are unwilling to bear [49,50]. At present, there are also experiments proving that the combination of amitriptyline and other analgesic drugs, such as pregabalin, may have a better effect [47,51]. Topical drug use did not change much in our study range, although many clinical trials have confirmed that local drug use has a certain therapeutic effect on PHN and has fewer adverse effects [52,53,54].

In the combination of drugs, we found that the frequency of mecobalamin, which does not belong to main treatment drugs, was high. Mecobalamin is a vitamin medicine and is the activated form of vitamin B12. A few studies shows that it not only has a good therapeutic effect on PHN, but can also relieve peripheral polynomialism, entrapment neuropathy and glossopharyngeal neuropathy [55,56]. A study showed that in four trials including 383 participants, the scores of the pain numerical scale in the vitamin B12 group decreased faster, compared with the placebo group. Vitamin B12 can improve the quality of life of patients with PHN and significantly reduce the number of patients using analgesics [57]. The combined use of mecobalamin seems to be justified and reasonable, but more relevant studies are also needed in the future to confirm the safety of its use and its impact on patients.

There are also several limitations to our study. First, the severity of PHN and clinical outcome were not measured and matched with the prescription. If the patient’s pain degree is included in future studies, hierarchical statistics on the drugs used could be better gathered. Second, the rationale of drug use was not assessed, due to the large number of prescriptions. Other comorbidities may cause some deflection among the statistical results. Although all patients with prescriptions are diagnosed with PHN, they also contain many drugs that might not be used to treat PHN. Finally, sampling bias may exist: although prescriptions were from many hospitals located in representative areas of China, primary care or non-hospital-based outpatient prescriptions are not included in our study. Therefore, in future research plans, the study of the correlation between the pain degree of patients and the corresponding drug selection should be included, and include more patient disease information, making it possible to better analyze the rationality of drug use.

## 5. Conclusions

The status and trends of pharmacological treatment for outpatients with PHN in China during a five-year period were analyzed in this study, and the yearly prescriptions and corresponding costs were both found to have increased. Gabapentin and pregabalin were the most frequently used drugs for PHN, which is in accordance with current practice guidelines. Among them, the use and cost of pregabalin showed a significant increasing trend. Oxycodone, as an opioid drug with a strong analgesic effect, had the third most yearly prescriptions but took the largest share of cost, which raised concerns about the rationality of its use and economic burden for PHN patients. The discovery of mecobalamin as the most commonly used drug may be due to its beneficial effect on peripheral nerves, but more research is still needed to study its mechanism of action on PHN in future. The percentages of antidepressants and dermal drugs were relatively low, which reflected physicians’ behavior and patients’ preferences in China. The results of this study indicate that the relevant departments and prescribers should attach great importance to the use of drugs in the treatment of PHN, especially with regard to the use of addictive drugs. Our study may benefit the allocation of medical resources and management for PHN in China, as well as other countries.

## Figures and Tables

**Table 1:** Demographic characteristics of the study sample.

		2015	2016	2017	2018	2019	P1	P2
Age (year)	≤45	204 (8.1%)	258 (8.4%)	231 (7.3%)	333 (7.6%)	404 (7.9%)	0.086	0.424
	46–65	910 (36.1%)	1135 (37.1%)	1166 (37.0%)	1600 (36.5%)	1883 (36.7%)	0.027	0.970
	66–80	1009 (40.1%)	1221 (39.9%)	1280 (40.6%)	1766 (40.3%)	2035 (39.7%)	0.027	0.432
	≥81	396 (15.7%)	449 (14.7%)	478 (15.2%)	681 (15.5%)	807 (15.7%)	0.027	0.711
Sex	Male	1212 (48.1%)	1418 (46.3%)	1539 (48.8%)	2160 (49.3%)	2554 (49.8%)	0.027	0.198
	Female	1307 (51.9%)	1645 (53.7%)	1616 (51.2%)	2220 (50.7%)	2575 (50.2%)	0.086	0.115

Note: Data were presented as numbers (percentage%); P1: *p* value for trends in number in yearly prescriptions, assessed by the Mann–Kendall test; (P): P2: *p* value for trend in portion of prescriptions, assessed by the log-linear test.

**Table 2:** Number of yearly prescriptions for specific drugs from 2015 to 2019.

Drug Class	Name	2015	2016	2017	2018	2019	P1	P2
Anticonvulsant	Gabapentin	1053 (41.6%)	1405 (45.9%)	1492 (46.3%)	1792 (38.1%)	1651 (29.1%)	0.086	0.177
	Pregabalin	95 (3.7%)	108 (3.5%)	177 (5.5%)	848 (18.1%)	1413 (24.9%)	0.027	0.007
	Oxcarbazepine	25 (1.0%)	27 (0.9%)	21 (0.7%)	22 (0.5%)	36 (0.6%)	0.807	0.020
	Carbamazepine	36 (1.4%)	29 (0.9%)	35 (1.1%)	30 (0.6%)	28 (0.5%)	0.221	0.036
Opioid	Oxycodone	208 (8.2%)	342 (11.2%)	322 (10.0%)	586 (12.5%)	790 (13.9%)	0.086	0.031
	Tramadol	260 (10.3%)	354 (11.6%)	363 (11.3%)	443 (9.4%)	472 (8.3%)	0.027	0.184
	Codeine	157 (6.2%)	178 (5.8%)	223 (6.9%)	269 (5.7%)	204 (3.6%)	0.221	0.235
	Morphine	14 (0.6%)	35 (1.1%)	54 (1.7%)	218 (4.6%)	208 (3.7%)	0.086	0.070
Antidepressant	Amitriptyline	108 (4.3%)	133 (4.3%)	96 (3.0%)	144 (3.1%)	93 (1.6%)	0.807	0.035
	Doxepin	57 (2.2%)	44 (1.4%)	44 (1.4%)	39 (0.8%)	37 (0.7%)	0.043	0.007
	Duloxetine	33 (1.3%)	46 (1.5%)	51 (1.6%)	91 (1.9%)	159 (2.8%)	0.027	0.013
	Venlafaxine	7 (0.3%)	16 (0.5%)	19 (0.6%)	22 (0.5%)	16 (0.3%)	0.312	0.270
Topical formulation	Lidocaine	62 (2.4%)	61 (2.0%)	65 (2.0%)	109 (2.3%)	145 (2.6%)	0.086	0.462
	Capsaicin	59 (2.3%)	67 (2.2%)	87 (2.7%)	59 (1.3%)	55 (1.0%)	0.613	0.166
	Flurbiprofen	18 (0.7%)	35 (1.1%)	34 (1.1%)	68 (1.4%)	61 (1.1%)	0.221	0.222
	Diclofenac Sodium	42 (1.7%)	33 (1.1%)	35 (1.1%)	22 (0.5%)	21 (0.4%)	0.086	0.009

Note: Data were presented as numbers (percentage%); Percentage refers to the ratio of total PHN prescriptions in the current year; All drugs include their compound preparations; P1: *p* value for trends in number in yearly prescriptions, assessed by the Mann-Kendall test; P2: *p* value for trend in portion of prescriptions, assessed by the log-linear test.

**Table 3:** Yearly cost of specific drugs from 2015 to 2019.

Drug Class	Name	2015	2016	2017	2018	2019	P1	P2
Anticonvulsant	Gabapentin	155,505 (17.3%)	159,436 (16.2%)	151,208 (15.2%)	180,982 (8.8%)	195,228 (7.9%)	0.221	0.027
	Pregabalin	27,771 (3.1%)	32,793 (3.3%)	59,028 (5.9%)	290,640 (14.1%)	512,669 (20.8%)	0.027	0.002
	Oxcarbazepine	4357 (0.5%)	4900 (0.5%)	3098 (0.3%)	3774 (0.2%)	6248 (0.3%)	0.807	0.068
	Carbamazepine	1267 (0.1%)	944 (0.1%)	1299 (0.1%)	1149 (0.1%)	1278 (0.1%)	0.807	0.091
Opioid	Oxycodone	170,930 (19.0%)	204,129 (20.7%)	179,090 (18.0%)	526,434 (25.5%)	569,162 (23.1%)	0.086	0.220
	Tramadol	40,023 (4.5%)	41,393 (4.2%)	37,204 (3.7%)	47,173 (2.3%)	53,565 (2.2%)	0.221	0.015
	Codeine	11,925 (1.3%)	13,011 (1.3%)	12,002 (1.2%)	17,951 (0.9%)	14,457 (0.6%)	0.221	0.040
	Morphine	10,735 (1.2%)	18,246 (1.9%)	30,230 (3.0%)	90,688 (4.4%)	73,661 (3.0%)	0.086	0.161
Antidepressant	Duloxetine	7801 (0.9%)	10,319 (1.0%)	11,132 (1.1%)	18,926 (0.9%)	31,881 (1.3%)	0.027	0.215
	Venlafaxine	1282 (0.1%)	2302 (0.2%)	2760 (0.3%)	3498 (0.2%)	3590 (0.1%)	0.027	0.996
	Amitriptyline	785 (0.1%)	1113 (0.1%)	745 (0.1%)	1261 (0.1%)	1296 (0.1%)	0.221	0.084
	Doxepin	147 (<0.1%)	172 (<0.1%)	266 (<0.1%)	135(<0.1%)	114 (<0.1%)	0.462	0.669
Topical formulation	Flurbiprofen	10,396 (1.2%)	11,686 (1.2%)	6887 (0.7%)	14,361 (0.7%)	10,129 (0.4%)	1.000	0.022
	Lidocaine	4590 (0.5%)	2918 (0.3%)	3922 (0.4%)	7673 (0.4%)	15,481 (0.6%)	0.221	0.442
	Capsaicin	3888 (0.4%)	3868 (0.4%)	5069 (0.5%)	3551 (0.2%)	3413 (0.1%)	0.221	0.188
	Diclofenac Sodium	2217 (0.2%)	1490 (0.2%)	1358 (0.1%)	868 (<0.1%)	826 (<0.1%)	0.027	0.008

Note: Data were presented as costs (percentage%); percentage refers to the ratio of total PHN prescriptions’ costs in the current year; all drugs include their compound preparations; P1: *p* value for trends in costs in yearly prescriptions, assessed by the Mann–Kendall test; P2: *p* value for trend in portion of prescriptions’ costs, assessed by the log-linear test.

## Data Availability

The original contributions in the research are included in the articles, and further inquiries can be directed to the corresponding authors.
